# Encoding Cortical Dynamics in Sparse Features

**DOI:** 10.3389/fnhum.2014.00338

**Published:** 2014-05-23

**Authors:** Sheraz Khan, Julien Lefèvre, Sylvain Baillet, Konstantinos P. Michmizos, Santosh Ganesan, Manfred G. Kitzbichler, Manuel Zetino, Matti S. Hämäläinen, Christos Papadelis, Tal Kenet

**Affiliations:** ^1^Athinoula A. Martinos Center for Biomedical Imaging, Massachusetts General Hospital/Harvard Medical School/Massachusetts Institute of Technology, Charlestown, MA, USA; ^2^McGovern Institute, Massachusetts Institute of Technology, Cambridge, MA, USA; ^3^Department of Neurology, Massachusetts General Hospital, Harvard Medical School, Boston, MA, USA; ^4^Aix Marseille Université, CNRS, ENSAM, Université de Toulon, LSIS UMR 7296, Marseille, France; ^5^Montreal Neurological Institute, McGill University, Montreal, QC, Canada; ^6^Behavioural and Clinical Neuroscience Institute, University of Cambridge, Cambridge, UK; ^7^BabyMEG Facility, Fetal-Neonatal Neuroimaging and Developmental Science Center, Boston Children’s Hospital, Harvard Medical School, Boston, MA, USA; ^8^Division of Newborn Medicine, Boston Children’s Hospital, Harvard Medical School, Boston, MA, USA

**Keywords:** motion field, optical flow, MEG source imaging, Helmholtz–Hodge decomposition, epilepsy

## Abstract

Distributed cortical solutions of magnetoencephalography (MEG) and electroencephalography (EEG) exhibit complex spatial and temporal dynamics. The extraction of patterns of interest and dynamic features from these cortical signals has so far relied on the expertise of investigators. There is a definite need in both clinical and neuroscience research for a method that will extract critical features from high-dimensional neuroimaging data in an automatic fashion. We have previously demonstrated the use of optical flow techniques for evaluating the kinematic properties of motion field projected on non-flat manifolds like in a cortical surface. We have further extended this framework to automatically detect features in the optical flow vector field by using the modified and extended 2-Riemannian Helmholtz–Hodge decomposition (HHD). Here, we applied these mathematical models on simulation and MEG data recorded from a healthy individual during a somatosensory experiment and an epilepsy pediatric patient during sleep. We tested whether our technique can automatically extract salient dynamical features of cortical activity. Simulation results indicated that we can precisely reproduce the simulated cortical dynamics with HHD; encode them in sparse features and represent the propagation of brain activity between distinct cortical areas. Using HHD, we decoded the somatosensory N20 component into two HHD features and represented the dynamics of brain activity as a traveling source between two primary somatosensory regions. In the epilepsy patient, we displayed the propagation of the epileptic activity around the margins of a brain lesion. Our findings indicate that HHD measures computed from cortical dynamics can: (i) quantitatively access the cortical dynamics in both healthy and disease brain in terms of sparse features and dynamic brain activity propagation between distinct cortical areas, and (ii) facilitate a reproducible, automated analysis of experimental and clinical MEG/EEG source imaging data.

## 1 Introduction

MEG and EEG are the most direct correlates of neural currents measured externally (Baillet et al., 2001). Recent advances in both hardware and software (i.e., increase in number of sensors, faster microprocessors, and more accurate cortical surface reconstructions) have led to significant enhancement in the temporal and spatial resolution of these methods, which can now reach, sub-millisecond and sub-centimeter levels respectively (Murray et al., 2008; Papadelis et al., 2009). MEG and EEG measure the magnetic and electric correlates of intra-cranial currents respectively. In order to estimate the location and time-course of these neural current generators, we need to solve an ill-posed and non-unique inverse problem. The non-uniqueness of the inverse problem is a result of the non-triviality in the quasi-static Helmholtz equation that links the intra-cranial current sources to the observed extra-cranial fields. Spatiotemporal distributed source solutions, like minimum-norm estimate (MNE), of MEG/EEG have been proposed to overcome this non-triviality (Dale and Sereno, 1993; Hämäläinen and Ilmoniemi, 1994). The resulting current distribution incorporates the anatomical information for each individual brain from the magnetic resonance imaging (MRI), and calculates the time-course of source distributions usually constrained to the cortex. These MNE solvers leads to spatiotemporal linear solutions, and have been extensively used in the neuroimaging community for their relatively accurate source localizations and robustness to the noise levels normally present in MEG/EEG data sets.

This type of analysis leads to a huge amount of high-dimensional data containing large information in both time and space. Current approaches normally relies on studying cortical current variations at selected short latencies or by subtracting experimental conditions (e.g., standard minus deviant) to find features of interest in both space and time. These approaches allow mapping the local traveling of spatiotemporal cortical current activations on the cortical manifold. This propagation of brain activity via surface connections may represent propagating waves of cortical activity, which can emerge, transverse, or contract on the cortical surface (Ermentrout and Kleinfeld, 2001; Roxin et al., 2005; Gramfort et al., 2011).

The extraction of salient features mapping the spatiotemporal propagation of brain activity across different cortical regions relies so far on the expertise of neuroscience investigators or clinicians, who visually identify and quantify these patterns by using statistical tools. However, this procedure remains problematic since the statistical models are prone to subjective bias of the investigator. There is a definite need for methods that allow the automatic and *a priori* extraction of features of interest in evolving cortical dynamics and mapping in an automatic fashion the propagating activity between different cortical regions.

In this paper, we propose a novel method to overcome these difficulties using mathematical formalism we described previously (Lefèvre and Baillet, 2008; Khan et al., 2011). Our method extracts the spatiotemporal dynamics of EEG/MEG cortical sources using a combination of 2-Riemannian optical flow and Helmholtz–Hodge decomposition (HHD) on a highly-curved cortical manifold. The optical flow is a computer vision technique that represents the apparent motion in the time series of images. The HHD can automatically extract salient features from the optical flow.

Mathematically; HDD decomposes optical flow into:

- a non-rotational component deriving from the gradient of a scalar potential *U*;
- a non-diverging component deriving from the rotational of a scalar potential *A* (resp. vectorial potential, in 3D); and
- an harmonic part **H**, i.e., whose Laplacian vanishes.

In HHD, formalism features of interest are represented as critical points of scalar field *U* and *A*. Finding features as critical points in global field potential is much less sensitive to noise in the data and therefore less likely to get false positives (Khan, 2010). In comparison to current density, its spatiotemporal divergent component *U* yields more focal and compact representation of the cortical activity (Slater et al., 2008; Khan et al., 2011). In order to estimate the spatiotemporal propagation of brain activity, we should initially estimate and extract the features of interest using the diverging component *U*. Subsequently, HHD’s harmonic part **H** can infer how the information propagates between cortical areas, by a vector field which is both irrotational and incompressible. This Laplacian vector field can explain causal effects exerted by one brain region onto another. Particularly this vector field is especially applicable in revealing dynamics, which occur briskly in time and over short distances on the cortical manifold.

The detection of features in optical flow motion field using HHD has already been applied in many different imaging fields (Palit et al., 2005; Guo et al., 2006). For instance, it is used in cardiac video analysis, to detect features in cardiac motion fields that reflect pathological activity in the dynamics of cardiac electrical activity. The proposed method is based on previous work done by our group (Lefèvre and Baillet, 2008) where we introduced a variational method to estimate the optical flow on non-flat surfaces using a Riemannian formulation.

This previously proposed technique was used to analyze the global dynamics of cortically-distributed source images obtained from MEG or EEG data with also limited quantification of local dynamics (Lefèvre and Baillet, 2009). It was recently extended by introducing a new formalism to detect local features of the optical flow of cortical dynamics using a modified and extended approach to HHD (Slater et al., 2008; Khan et al., 2011).

This paper is structured as following: the Riemannian framework for optical flow on non-flat surfaces is first briefly introduced. The HHD formalism on 2-Riemannian manifolds will be presented next. Lastly, the application of HHD on simulated and human MEG data will be presented. The methods discussed in this paper are implemented in MatLab and are available for download as plugin to Brainstorm (MEG/EEG data processing software) (Tadel et al., 2011) at http://neuroimage.usc.edu/brainstorm. These methods will also soon be available for MNE–Python framework (Gramfort et al., 2014).

## 2 Materials and Methods

The HHD-based feature detection technique consists of three distinct steps.

- First the optical flow of distributed MEG/EEG MNE estimates is computed on the cortical manifold. In Section 2.1, we will briefly introduce optical flow and its mathematical formulation.
- In the second step, HHD is applied on optical flow computed previously. We will present HHD framework in Section 2.2 and concisely describe its axioms.
- Lastly, detecting the feature of interest in cortical dynamics now becomes the simple problem of identifying critical points in HHD scalar potential *U*. The traveling cortical dynamics can be tracked by vectors having highest norm in vector field *H* (see Section 2.3 for details).

### 2.1 Optical flow

We have introduced the concept of optical flow on a 2-Riemannian manifold (Lefèvre and Baillet, 2008), and we shall briefly summarize the approach as follows. Under the seminal hypothesis of the conservation of a scalar field *I* along streamlines, defined on a surface ℳ, the *optical flow* **V** is a vector field that satisfies:

(1) $$\partial_{t}I+g\left(V,\nabla_{M}I\right)=0.$$

Note that the scalar product *g*(.,.) is modified by the local curvature of ℳ, the domain of interest. The solution to Equation (1) is not unique as long as the components of **V**(*p, t*) orthogonal to ∇_ℳ_I are left unconstrained. This so-called “aperture problem” has been addressed by a large number of methods using e.g., regularization approaches. These latter approaches may be formalized as the minimization problem of an energy functional, which both includes the regularity of the solution and the agreement to the model:

(2) $$E\left(V\right)=\int_{M}\left[\right.\frac{\mathrm{\partial I}}{\mathrm{\partial t}}+g\left(V,\nabla_{M}I\right){]}^{2}\mathrm{d\mu}+\lambda \int_{M}C\left(V\right)\mathrm{d\mu}.$$

Here, we considered a regularity factor operating quadratically on the gradient of the expected vector field:

(3) $$C\left(V\right)=Tr{(}^{t}\nabla V.\nabla V).$$

Note that in order to be an intrinsic tensor, the gradient of a vector field must be defined as the covariant derivative associated to the manifold ℳ. Due to space constraints, we need to refer to essential elements of differential geometry for more information on this notion (Do Carmo, 1993).

### 2.2 Helmholtz–Hodge decomposition on 2-Riemannian manifold

Let us consider ℳ as a surface (or manifold) parameterized by local charts (*x*_1_, *x*_2_). It is thus possible to obtain a normal vector at each surface point:

$$n_{p}=\frac{\partial }{\partial x_{1}}\wedge \frac{\partial }{\partial x_{2}}.$$

Note that the normal vector does not depend on the choice of the parameterization (*x*_1_, *x*_2_). We then define the gradient and divergence operators through duality:

$$\begin{array}{llll} \text{d}U\left(V\right) & =g\left(\nabla_{M}U,V\right),\; & & \;\\ \int_{M}\;U{\text{ div}}_{M}H & =-\int_{M}\;g\left(H,\nabla_{M}U\right).\; & & \; \end{array}$$

Finally, scalar and vectorial curl operators are obtained through:

$$\begin{array}{llll} {\text{Cu}}_{M}A & =\nabla_{M}A\wedge n,\; & & \;\\ {\text{cu}}_{M}H & ={\text{ div}}_{M}\left(H\wedge n\right).\; & & \; \end{array}$$

Again, these formulas are intrinsic expressions that do not depend on the parameterization of the surface.

Let us then reformulate results established in Polthier and Preuss (2003) for Riemannian manifolds. Given **V**, a vector field in Γ^1^(ℳ), there exists unique functions *U* and *A* in *L*^2^(ℳ) and a vector field **H** in Γ^1^(ℳ) such that:

(4) $$V=\nabla_{M}U+{\text{Cu}}_{M}A+H,$$

with

$$\begin{array}{cc} {\text{cu}}_{M}\left(\nabla_{M}U\right)=0,\; & {\text{div}}_{M}\left({\text{Cu}}_{M}A\right)=0,\\ {\text{div}}_{M}H=0,\; & {\text{cu}}_{M}H=0. \end{array}$$

Following classical constructions, *U* and *A* minimize the two following functionals:

$$\begin{array}{rcll} \int_{M}||V-\nabla_{M}U|{|}^{2}, & & \;\int_{M}||V-{\text{Cu}}_{M}A|{|}^{2}, & \end{array}$$

where ||⋅|| is the norm associated to the Riemannian metric *g*(.,.). These two functionals are convex and therefore have a minimum on *L*^2^(ℳ) satisfying:

(5) $$\begin{array}{ll} \forall \phi \in L^{2}\left(M\right),\;\int_{M}g\left(V,\nabla_{M}\phi \right) & =\int_{M}g\left(\nabla_{M}U,\nabla_{M}\phi \right), \end{array}$$

(6) $$\begin{array}{ll} \forall \phi \in L^{2}\left(M\right),\;\int_{M},g\left(V,{\text{Cu}}_{M}\phi \right) & =\int_{M}g\left({\text{Cu}}_{M}A,{\text{Cu}}_{M}\phi \right). \end{array}$$

Through (ϕ_1_, …, ϕ_n_) as basis functions, we may write **U** = (*U*_1_, …, *U_n_*)*^T^*, **A** = (*A*_1_, …, *A_n_*)*^T^*, and Equations (5) and (6) read, using array notations:

(7) $$\begin{array}{ll} \left[\right.\int_{M}g\left(\nabla_{M}\phi_{i},\nabla_{M}\phi_{j}\right){]}_{i,j}U & =\left[\right.\int_{M}g\left(V,\nabla_{M}\phi_{i}\right){]}_{i}, \end{array}$$

(8) $$\begin{array}{ll} \left[\right.\int_{M}g\left({\text{Cu}}_{M}\phi_{i},{\text{Cu}}_{M}\phi_{j}\right){]}_{i,j}A & =\left[\right.\int_{M}g\left(V,{\text{Cu}}_{M}\phi_{i}\right){]}_{i}. \end{array}$$

The harmonic component **H** of vector field **V** is simply obtained through:

(9) $$H=V-\nabla_{M}U-{\text{Cu}}_{M}A.$$

### 2.3 Feature detection as critical points of HHD potentials

The critical points of a vector field are often classified depending on the eigenvalues of the Jacobian matrix defined locally in a vector field. In our case, however, critical points of the flow can be found as local extrema of the potentials *A* and *U*. Finding features as critical points on global potential fields is much less sensitive to noise in the data and therefore is less susceptible to false positives, than with methods using local Jacobian eigenvalues (Tong et al., 2003). Moreover unlike eigenvalues methods, HHD do not pre-specify the number of features.

A sink (resp. a source) corresponds to a local minimum (resp. maximum) of *U*. Similarly, a counter-clockwise (resp. clockwise) vortex may be detected through a local minimum (resp. maximum) of *A*. Detection of traveling cortical activity on the surface can be performed by tracking vector **H** having largest norm.

## 3 Results

We will now present the application of HHD on one simulated and two actual MEG datasets. In Section 3.1 under a simulated scenario, HHD is used to track and encode cortical activity as it emerges from the somatosensory cortex, traveling along the central sulcus, and receding in the inferior frontal gyrus. The first MEG dataset presented in Section 3.2 is obtained from a median nerve stimulation paradigm that consists of a train of electrical stimuli applied on the wrist of a healthy adult individual. It is a typical experimental paradigm to elicit activity within the primary somatosensory cortex. The second MEG dataset shown in Section 3.3 is from a pediatric epilepsy patient with tuberous sclerosis complex. In this dataset, we track the propagation of the epileptogenic activity within the irritative zone.

### 3.1 Simulation data

The use of HHD to address the quantitative and qualitative evaluation of this technique is illustrated below.

#### 3.1.1 Generation

**Figure 1:**
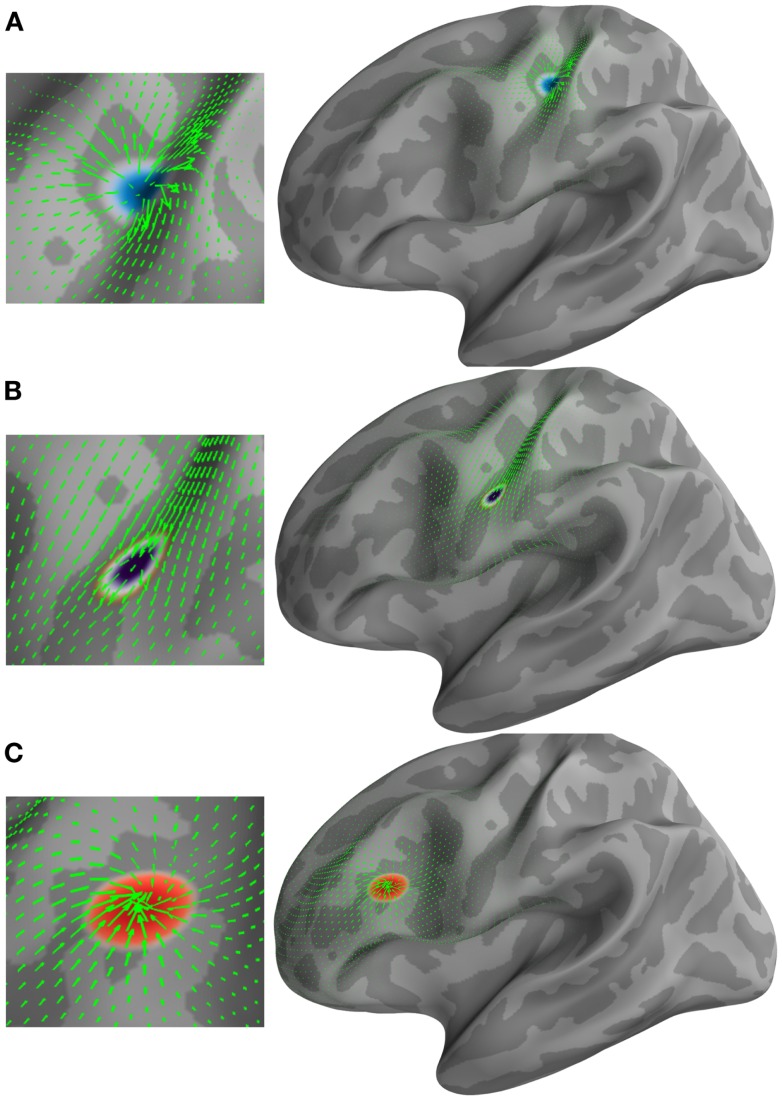
**Examples of different types of vector fields (green arrows) and their corresponding *U* and (*H*) HHD components**. **(A)** Emerging source represented by a minima in the scalar potential *U*. **(B)** Traveling source detected by (*H*). **(C)** Receding source represented by a maxima in the scalar potential *U*.

The brain surface from Freesurfer’s FsAverage was selected to demonstrate the applicability of the method on the cortical manifold. This surface consisted of 10,242 vertices and 20,480 triangles. A source on this manifold was generated in the vicinity of the central sulcus, also known as the primary somatosensory area (S1). This source was grown to a patch of 5 cm^2^ (geodesic area) in five time steps. Subsequently, a constant vector field was defined in time from the vectorial heat equation. An advection equation (Lefèvre and Baillet, 2008) was used to transverse this patch on the manifold. Finally, this patch was contracted in five time steps in the vicinity of the inferior frontal gyrus. The results of this simulation are presented in Figure 1. The three stages of the source evolution are shown as Figures 1A–C.

#### 3.1.2 Analysis

We first applied the optical flow on this simulated activity. Optical flow transformed the dynamics of the source’s evolution in terms of the motion vector fields that were emerging, traversing, and receding. This optical flow computation was performed in the MNE–Python framework and took 10.75 s to compute. We then applied HHD to the optical flow at each time step, which detects three main features corresponding to the three stages of the simulation. HHD took 30.36 s to decompose optical flow in the discrete feature set. The simulation was performed on a workstation having a Dual Octa Core Xeon CPUs (32 Threads) with 64 GB of RAM.

#### 3.1.3 Results

Figure 1 shows the applicability of HHD on the distributed current density maps for three different source configurations: (A) an emerging source; (B) a moving source having constant velocity; and (C) a descending source (sink). HHD automatically detects these three features from optical flow as critical points in *U* and **H**. A source represented by a minima in the scalar potential *U* (texture colormap in blue) is shown in Figure 1A. The vector field (green arrows) represents optical flow computed earlier. It is indicated in Figure 1A that HHD is able to capture the growing dynamics of the cortical activity. In Figure 1B, traveling activity is tracked by the highest norm vectors in field **H**. The texture colormap represents the norm of *H*, where the highest norm is shown with the dark violet texture map. In Figure 1C, receding cortical dynamics are captured by a maxima in the scalar potential *U*. This texture colormap represents *U* with a maxima shown in red. Again, the vector field is shown with green arrows representing optical flow.

### 3.2 Feature analysis of experimental MEG somatosensory data

#### 3.2.1 Experiment

MEG data was recorded from a 28-year-old healthy female individual at the MEG laboratory of the Center for Mind/Brain Sciences (CIMeC), University of Trento, Italy. During the experiment, the median nerves of her right and left wrists were electrically stimulated transcutaneously. Approval was obtained from the University of Trento Ethics Committee, Italy, and the subject gave her written informed consent before the experiment. Constant current stimuli with a duration of 0.2 ms, and a pseudo-randomized inter-stimulus interval of 250 ± 50 ms were applied to the subject. Before the start of the experiment, we measured two basic intensity thresholds for each of the subject’s wrists. The two parameters were the motor threshold (MTH), defined as the minimal stimulus intensity needed to produce thumb twitching, and the sensory threshold (STH), defined as the minimal stimulus intensity at which the subject was just able to feel a train of stimulus pulses. The stimuli were delivered either to the right or to the left wrist with two intensity levels of *M* = *MTH* + 0.25 Δ and *S* = *STH* + 0.25 Δ, where Δ = *MTH* − *STH*. Here, we used only the data from the right median nerve stimulation and the *M* intensity level. More details about the experimental design can be found in Papadelis et al. (2012).

Somatosensory evoked fields (SEFs) were recorded at a sampling rate of 5 kHz by using a 306-channel (204 first order planar gradiometers, 102 magnetometers) VectorView MEG system (Elekta-Neuromag Ltd., Helsinki, Finland) placed inside a magnetically shielded room (AK3B, Vacuumschmelze, Hanau, Germany). Hardware filters were adjusted to band-pass the MEG signal in the frequency range of 0.01–1000 Hz. Data from 200 trials were used in this study.

#### 3.2.2 Data analysis

**Figure 2:**
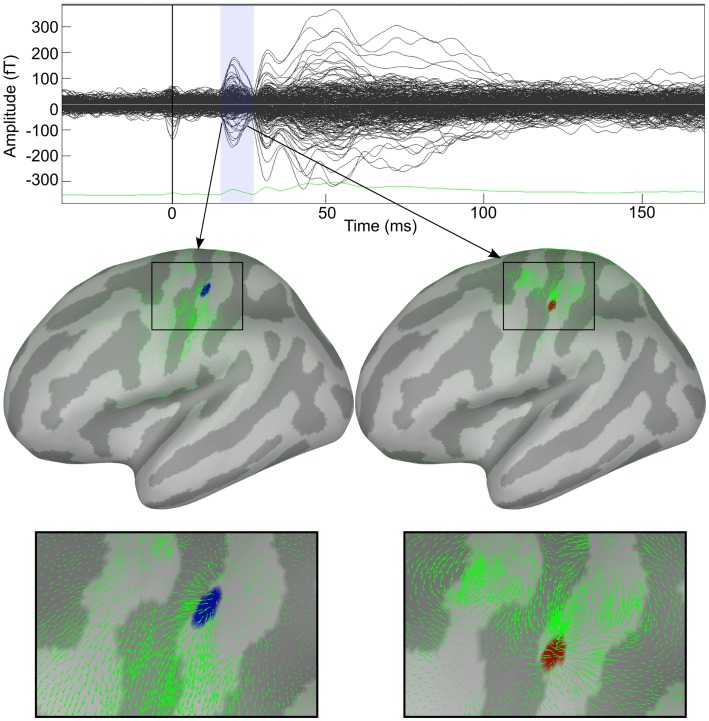
**Decomposition of cortical activity in a series of dynamic features**. Response to median nerve stimulation of the right wrist. Upper panel shows the butterfly plot of somatosensory evoked fields (SEFs). Two features identified by HHD are shown in the middle panel. Bottom panel shows zoom view of the detected features.

To compensate for head movements during the measurements and suppressing external magnetic disturbances, the signal space separation (SSS) algorithm (Taulu and Simola, 2006), as implemented with the MaxFilter software (Elekta-Neuromag), was performed offline on the raw MEG data. The corrected MEG data were then filtered offline in the band of 0.1–150 Hz and epoched from −50 to 200 ms relative to the stimulus onset. Trials contaminated with artifacts were excluded from further processing. In total, 160 trials survived the rejection criteria. These trials were baseline corrected and then averaged. The subject’s MRI was processed using FreeSurfer; cortical and head surfaces were extracted. Cortical surface was downgraded to 50,003 points and elementary current dipoles were positioned at the surface of the cortex of the subject. The multi-sphere forward model was computed and the standard minimum-norm was used to estimate cortical currents. The pre-processing and generation of both the forward and inverse solution was done in Brainstorm (Tadel et al., 2011). The MNE solution was computed at every point within the time window that represents the N20 component (from 15 to 25 ms) (see Figure 2, upper panel). The N20 component represents the first cortical response to the electrical stimulation of the median nerve. Optical flow was estimated from the minimum-norm data. HHD was then applied on this optical flow to detect sparse features in cortical dynamics. The computation of optical flow took 50.33 s whereas HHD took 123.73 s on the workstation mentioned in Section 3.1.2. This application was done in the MatLab implementation of the HHD.

#### 3.2.3 Results

Figure 2 shows the applicability of HHD on the distributed source maps of MEG data recorded during the median nerve stimulation experiment. Our method reveals the diverging and contracting cortical mechanisms in the primary somatosensory cortex. Brain responses during N20 were automatically decomposed into two features: a source and a sink.

In Figure 2, the upper panel shows the butterfly plot of somatosensory evoked fields (SEFs). Two features identified by HHD are shown in the middle panel of Figure 2; an emerging source at 17.3 ms (MNI: −46.74, −30.17, 66.84) and a descending source (sink) at 23.2 ms (MNI: −55.86, −22.26, 51.20). The green arrows represent the motion field of the cortical dynamics as estimated by the optical flow. HHD reveals that the first cortical responses appear at the top of S1 in the central sulcus, travels down the sulcus, and then sinks down at lower edge of the sulcus. The bottom panel presents a zoom up view of these two features. HHD reduces the size of the dataset from 50,003 × 50 (spatial dimension × temporal dimension) to two main features. This example presents the concept of our methodology to obtain a compact representation of cortical activity during a cognitive experiment.

### 3.3 HHD characterization of epileptic activity

Our second application of HHD is on MEG data acquired from a subject having clinical history of refractory epilepsy. In this application, we will focus on the property of the *U* component of HHD, which allows for the detection of activity that changes rapidly in both time and space.

#### 3.3.1 Epileptic data

**Figure 3:**
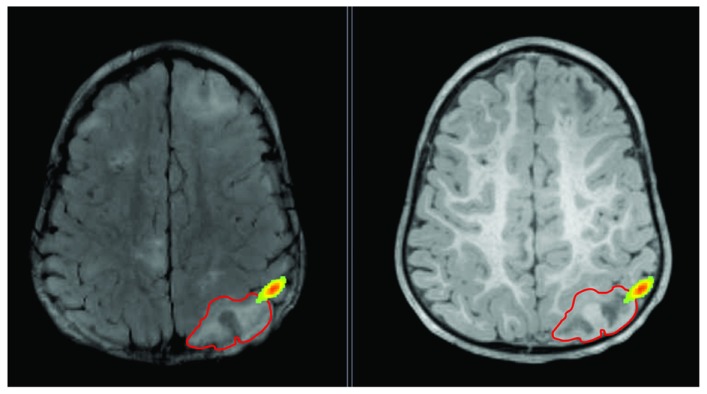
**Lesion is shown on T2 (left) and T1 (right) with red outline**. Epileptic activity emerging from the edge of the lesion is shown as texture map.

The data was recorded from a 20-month-old boy, who presented his first seizure at the age of 3 months, with refractory epilepsy as a result of TSC. The patient had multiple subcortical tubers identified on his MRI as patchy areas of T2 prolongation, stable over time (see Figure 3). Long-term monitoring revealed 54 seizures in total (46 electroclinical and 8 electrographic seizures) with duration of 10–44 s. The seizure onset was localized at the right posterior quadrant (electrodes P8, O2, P4, T8, and C4). Routine and ambulatory EEG has indicated frequent interictal sharp waves at electrodes C4, Pz, P4, and P8, as well as slowing at the right posterior quadrant.

MEG data were recorded for 45 min during sleep at the BabyMEG facility located at the Radiology Suite of Boston’s Children Hospital (Waltham, MA, USA). MEG recordings were performed using a 74-sensor MEG system especially designed for pediatric use (“babySQUID” – Tristan Technologies Inc., San Diego, CA, USA). The babySQUID system is accommodated in a single-layer magnetically shielded room (MSR). MEG data was sampled at 1024 samples per second. The sensor array of MEG was covering the right posterior quadrant. T1-weighted high-resolution magnetization-prepared rapid gradient echo (MPRAGE) structural images were acquired on a 3.0-T Siemens Trio whole body MR scanner (Siemens Medical Systems, Erlangen, Germany) using a 32 channel head coil. Details about the experimental procedure can be found elsewhere (Papadelis et al., 2013). Research MEG and MRI data were acquired and analyzed after explicit parental consent under a protocol approval by the Boston’s Children Hospital institutional review board.

#### 3.3.2 Analysis

**Figure 4:**
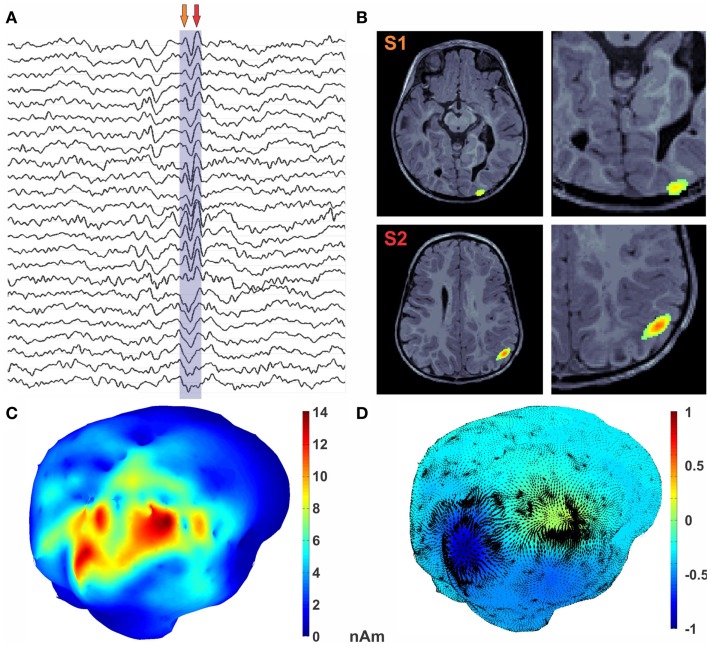
**Encoding of epileptic spike in diverging features**. **(A)** MEG traces with epileptic spike marked. **(B)** MRI with MEG activity represented as the color texture. **(C)** Average MEG activity during the spike. **(D)** *U* HHD scalar potential is mapped onto the cortical surface using textured colors. The divergence part of vector flow of MEG sources is represented by green arrows at each vertex location. Critical points in the *U* map (shown with magenta sphere) reveal sources shown in dark blue and sink in red.

A high number of interictal spikes (>20) were identified by a board-certified epileptologist with a consistent spatiotemporal pattern indicating a focal source in the right posterior quadrant. The MEG data was then filtered offline in the band of 0.1–145 Hz. The subject’s MRI was processed using brainvisa; cortical and head surfaces were extracted. To compute the forward solution, a boundary-element model (BEM) with a single compartment bounded by the skull’s inner surface was assumed (Hämäläinen et al., 1993). The watershed algorithm was used to generate the inner skull surface triangulations from the high-resolution T1 MR images of each participant. The current distribution was estimated using the MNE by fixing the source orientation to be perpendicular to the cortex. The noise covariance matrix used to calculate the inverse operator was estimated from empty-room data. In order to reduce the bias of the MNEs toward superficial currents, we incorporated depth weighting by adjusting the source covariance matrix (Lin et al., 2006). To estimate the epileptic foci for the subject from HHD, a single sharp epileptic spike (Figure 4A) was selected. MNE was then computed for this spike in both volume and cortical space. Optical flow was calculated from the MNE estimated cortical currents at each time point during a time window shown in Figure 4A. HHD was computed by decomposing this optical flow and extracting the diverging scalar field *U*. The computation of optical flow and HHD took 34.7 s on the workstation mentioned in Section 3.1.

#### 3.3.3 Results

In Figure 4B, MNE activity is presented in the volume at the two arrow points in Figure 4A. Average MNE activity on the cortical manifold at these two points is shown in Figure 4C. Critical points are then searched in the scalar field. This process results in finding two critical points in this time window, which corresponds to the signature features of this epileptic spike. A source in the posterior occipital region S1, from where the activity emerges, is shown in blue in Figure 4D. This activity sinks in the anterior parietal cortex in the vicinity of a subcortical tuber. This sinking activity is shown in red in Figure 4D. Finally, the diverging vector field ∇_M_U is computed by taking the gradient of the scalar field *U*. In Figure 4D, the divergence vector field for this epileptic spike is shown with black arrows whereas color texture represents strength of *U*.

## 4 Discussion

In this paper, we present the applicability of HHD to high-dimensional neuroimaging data. The HHD-based sparse feature encoding technique works in three steps. First, MNE is computed to estimate cortical current activity. Optical flow is then used to estimate the motion field of distributed cortical dynamics. Finally, the optical flow is decomposed into sparse and compact features using HHD and the neuroimaging feature extraction simplifies to the problem of finding critical points in the scalar potential *U* and by the highest norm vector of **H**.

The method has been optimized for running in multi-core CPUs in order to decompose high-dimensional MEG/EEG data (~GB) in few seconds on a highly dense (~50,000 vertices) cortical mesh. The method provides a compact representation of the cortical dynamics. The method also allows to track the cortical activity as it appears, disappears, or travels on the cortical manifold. Moreover, HHD can also infer the information flow between cortical regions over short distances and times. Here, we present the application of our method on both simulated and actual MEG data sets.

In the simulated scenario, we generated synthetic cortical activity from the heat vector field and the advection equation. We then applied optical flow and HHD on this simulated data compacting the different stages of these dynamics in three sparse features. These results were in absolute accordance with our generated data. In the cognitive neuroscience application, we encoded spatiotemporal time varying maps of cortical activity during electrical stimulation of the median nerve. We showed that HHD and optical flow can compress cortical dynamics of N20 component in two distinct features; a source and a sink. This is in accordance to recent evidence in the literature of somatosensory processing which demonstrate that the earliest cortical response component (N20) after median nerve stimulation is generated by two generators located within S1 subdivisions and being active following a serial fashion (Inui et al., 2004; Papadelis et al., 2011). The application of our method in neuroscience data may enable the reproducibility of cognitive experiments across different sessions or research centers.

Our method can significantly contribute to epilepsy research, because it is able to detect and map the propagation of interictal spikes across time. MEG and EEG studies have so far shown that these two neuroimaging methods can non-invasively detect the propagation of spikes in epilepsy patients (Sutherling and Barth, 1989; Emerson et al., 1995; Tanaka et al., 2010). We also like to emphasize the importance of initial detection of the spike with a consistent spatiotemporal pattern indicating a focal source by an epileptologist, as the method seeds from it.

The investigation of epileptic spike propagation is important for the understanding of the pathophysiology of epilepsy and for the appropriate clinical decision during the presurgical evaluation of epileptic patients. Spike propagation can reflect neural networks associated with epilepsy (Spencer, 2002), while the propagation pattern of interictal spikes has been shown to be related to the outcome of epilepsy surgery (Hufnagel et al., 2000; Schulz et al., 2000). In the application of HHD to epilepsy data, we exploited the sensitivity of the divergence component *U* to fast changes in space and time, such as these during a propagating interictal spike. The epileptic activity at the onset of the interictal spike can be sharp spatio-temporarily, and may easily be detected by HHD. We used HHD in deciphering these complex interictal spike dynamics. Using HHD, we identified where the spike initiated but also tracked its activity as it propagated on the cortical manifold. The application of HHD found two critical points on the cortical manifold, a source and a sink, both located at the abnormally developed tissue surrounding a tuber rather than the tuber itself. The epileptogenic activity was propagating across time along the borders of the tuber, and in any instance was crossing the tuber itself. Our findings are in agreement with previous studies indicating that in TSC patients with epilepsy the epileptogenic tissue is predominately localized in the surroundings of the cortical tubers (Weiner, 2004; Xiao et al., 2006; Major et al., 2009), and a single case study published in this issue (Hunold et al., 2014). The high sensitivity of our method allowed us to map the evolution of the epileptiform activity across time with respect to the location of the tuber. This critical, automatically extracted, spatiotemporal information of interictal spikes may provide more accurate information of spike propagation in epilepsy patients compared to the classical, observer-dependent methods, and thus it may be clinically useful in the presurgical evaluation of epilepsy patients.

## 5 Conclusion

We demonstrate the applicability of our HHD technique on high-dimensional electrophysiological data from neuroscience and clinical research data. Salient features of our technique, which are demonstrated by our results are: (i) sensitivity to spatiotemporal diverging cortical sources rapidly evolving in space and time (within few milliseconds), (ii) consideration of geometry of the cortical manifold on which the neural activity is evolving, (iii) automatic extraction of the spatiotemporal features, (iv) automatic characterization of the cortical activity propagation across different brain regions, (vi) visualization of the salient feature of cortical activity, and (vii) application in both cognitive and clinical neuroscience (i.e., propagation of epileptiform activity). Here, we present some of the possible applications of HHD in the neuroimaging field. We strongly believe that there are much more applications of our method in both neuroscience as well as clinical research. Further improvements of our method include: the discretization to higher-order finite element analysis, statistical analysis of the detected features, and the further reduction of the algorithm computational complexity. HHD is freely available as a plugin for major MEG processing suites (i.e., Brainstorm and MNE–Python) and readers are encouraged to use and extend the proposed method.

## Conflict of Interest Statement

The authors declare that the research was conducted in the absence of any commercial or financial relationships that could be construed as a potential conflict of interest.
